# Enhanced well-being in second language learners: unraveling the roles of emotion regulation and resilience

**DOI:** 10.3389/fpsyg.2025.1627834

**Published:** 2025-10-22

**Authors:** Hong Shi, Jiayao Gao

**Affiliations:** School of Foreign Languages, China University of Petroleum, Beijing, China

**Keywords:** emotion regulation, resilience, well-being, second language learners, positive psychology

## Abstract

With the growing interest in positive psychology, the roles of well-being, emotion regulation, and resilience in second language acquisition (SLA) have attracted increasing attention. Emotion regulation aids in maintaining positive emotions and managing study stress, while resilience helps individuals maintain a positive attitude and recover swiftly from difficulties. Both factors significantly impact the well-being of SLA learners. This study investigated the relationship among these variables using questionnaires on 157 English language learners from a top university in China. Correlation and regression analyses, conducted using SPSS, revealed significant positive correlations between emotion regulation (cognitive reappraisal and expressive suppression), resilience, and well-being. Resilience exhibited the strongest correlation with well-being, followed by its correlation with cognitive reappraisal and expressive suppression. Cognitive reappraisal had a significant positive predictive effect on well-being, with resilience partially mediating this relationship. On the other hand, expressive suppression indirectly affected well-being solely through resilience. The findings underscore the importance of enhancing emotion regulation skills and resilience to improve SLA learners’ well-being, providing insights for educational strategy development and learner support.

## Introduction

Well-being, a cherished goal and fundamental right, necessitates cultivation through education (Xu and Yang, 2023). Its importance in contemporary education policies underscores its role as a cornerstone for learner development and a reflection of educational quality (Mercer, 2021). In the realm of second language acquisition (SLA), well-being has emerged as a pivotal focus due to its correlation with optimal learner performance and resilience against stressors like academic pressure and cross-cultural challenges (Oxford, 2016).

Positive psychology has brought renewed attention to well-being among SLA learners, yet this perspective remains under-explored in the Chinese context (Xu, 2024). Within positive psychology, emotion regulation and resilience are crucial constructs influencing well-being. Emotion regulation, a component of resilience, fosters well-being by nurturing positive emotions (Braunstein et al., 2017).

Internationally, studies have delved into the relationships between well-being and its influencing factors, such as emotion regulation (Greenier et al., 2021) and resilience (Proietti Ergün and Dewaele, 2021). Emotion regulation enhances positive emotions, contributing to well-being (Braunstein et al., 2017), while resilience safeguards individuals from adversity’s adverse effects, promoting psychological well-being (Connor and Davidson, 2003). In China, research has shown that cognitive reappraisal, an emotion regulation strategy, positively impacts migrant children’s well-being (Chai et al., 2018).

Previous studies have suggested that resilience often functions as a mediator linking psychological or emotional resources to well-being. For example, Bajaj and Pande (2016) found that resilience partially mediated the effect of mindfulness on life satisfaction, while Akbari and Khormaiee (2015) demonstrated that resilience mediated the relationship between emotional intelligence and psychological well-being. These studies collectively highlight resilience as an adaptive psychological mechanism that transforms emotion regulation strategies into improved well-being outcomes. Building on this literature, we hypothesized that resilience would mediate the relationship between emotion regulation and well-being in the present study.

However, research on SLA learners’ well-being is nascent, with limited focus on emotion regulation and resilience (Li and Lv, 2022; Liu et al., 2022). Furthermore, studies on resilience’s mediating role in the relationship between emotion regulation and well-being are scarce.

English as a Second Language (ESL), as a prototypical group of SLA, learning presents a unique set of difficulties. L2 learners face the unique identity tensions, particularly the dual role of English as both a foreign language and a global lingua franca (Jenkins, 2015). Language learners often struggle with mastering complex grammar, building an extensive vocabulary, and achieving fluency. These challenges can trigger stress, anxiety, and self-doubt, directly affecting their emotion regulation. For example, when facing difficult language tasks like writing a complex essay or participating in a language-intensive discussion, learners may experience high levels of stress. Additionally, cross-cultural differences in English-speaking contexts can lead to culture shock, making it difficult for learners to adapt. This unique combination of factors makes the emotional states, resilience, and well-being of second-language learners distinct.

Against this backdrop, this study employs ESL learners as participants to fill this gap by investigating how emotion regulation and resilience impact the well-being of SLA learners. Although previous studies have examined emotion regulation and resilience separately, fewer have investigated them jointly. Exploring both constructs simultaneously allows us to clarify whether their effects on well-being are additive, overlapping, or mediated, which is critical for designing targeted educational interventions. ESL learners represent a large and diverse group in the global SLA context, and the insights gained from studying them can be widely applicable. By understanding these relationships, we can derive implications for enhancing learners’ language acquisition experiences and overall well-being. Based on its contribution to the existing literature, this study aims to provide foundational insights that can inform the development of interventions designed to enhance the well-being and progress of second language (L2) learners. The research questions guiding this inquiry are as follows:

1. What is the nature of the correlation among L2 learners’ emotion regulation, resilience, and well-being?
2. To what extent can L2 learners’ emotion regulation predict their well-being?
3. Does resilience mediate the relationship between emotion regulation and well-being among L2 learners?

## Literature review

### Well-being

With the advent of Positive Psychology, researchers have increasingly focused on enhancing positive emotions (Peterson, 2006). Oxford (2016) underscores the significance of positive psychology, emphasizing its focus on human well-being, which is often defined as the presence of positive emotions, engagement, and a sense of meaning in life (Seligman and Csikszentmihalyi, 2000). According to Seligman and Csikszentmihalyi (2000), the ultimate goal of Positive Psychology is to identify factors that contribute to human prosperity, mental health, and personal well-being. This foundation has led to the development of various models and measures of well-being that aim to enhance individual growth and societal development.

Ancient Greek philosophy offers two main perspectives on well-being: the hedonistic approach, which focuses on pleasure and desire, and the eudaimonic approach, which emphasizes self-actualization and flourishing (Garg et al., 2014). The eudaimonic perspective has been more widely adopted in contemporary well-being research, as it aligns with the long-term, meaningful aspects of life satisfaction. In this context, Seligman (2011) presents the PERMA model of well-being, which encompasses five core elements: Positive Emotion, Engagement, Relationships, Meaning, and Achievement. The model not only identifies key areas contributing to well-being but also suggests that these dimensions can be learned and cultivated over time (Fredrickson, 2001). This holistic approach has been particularly useful in understanding well-being in educational contexts (Butler and Kern, 2016).

For L2 learners, well-being involves deriving joy from language acquisition, active language learning engagement, supportive interactions with teachers and peers, finding purpose in learning, and a sense of accomplishment. These elements collectively promote overall well-being, creating a positive language learning experience (Seligman, 2011). The PERMA model has been proven significant in positive education (Butler and Kern, 2016; Wang et al., 2021) and aids in understanding and encouraging L2 learners’ well-being.

However, research predominantly focuses on individual dimensions of PERMA rather than considering its full, multidimensional application (Dewaele et al., 2019). This limited scope may hinder a comprehensive understanding of how well-being operates in L2 learning environments. To address this, future research must focus on a more integrated approach to well-being in L2 education that considers all five dimensions of the PERMA model, promoting holistic development for both learners and educators.

Recognizing and supporting the emotional and psychological well-being of instructors and students in L2 education is essential, as it fosters a conducive learning atmosphere and enhances positive academic and emotional experiences (Mercer, 2021). Research has shown that a heightened sense of well-being increases foreign language engagement (Proietti Ergün and Dewaele, 2021). Well-being also significantly impacts educators, enhancing their work engagement and emotion regulation proficiency, thereby creating a more enriched and satisfying teaching environment (Greenier et al., 2021). In addition, recent findings suggest that educator well-being is closely shaped by relational dynamics. Dillard-Cooke et al. (2025) demonstrate that students’ perceived similarity with their educators significantly influences educators’ well-being, indicating that well-being in educational settings is not only an individual trait but also constructed through interpersonal relationships.

### Emotion regulation

Emotion regulation has gained prominence in L2 education, driven by interest in positive psychology and the desire to understand the factors that influence L2 learners’ emotional and cognitive development (Fredrickson, 2001). Gross (1998) posits that emotions arise from repeated attention-reaction situations and can influence cognition and behavior. In L2 learning, emotion regulation significantly impacts educational outcomes, shaping learners’ experiences and overall success in acquiring a second language (Bielak and Mystkowska-Wiertelak, 2022).

Emotion regulation has been linked to a variety of academic factors in L2 education, including motivation, self-efficacy, engagement, burnout, and mindfulness (Kim and Kim, 2017; Li, 2023; Liu et al., 2022; Namaziandost et al., 2023). Research consistently indicates that the ability to regulate emotions enhances academic engagement and performance, with emotion regulation serving as a key predictor of success in language learning. This is particularly important because L2 learners often face emotional challenges such as anxiety, frustration, and stress during the language acquisition process.

Studies on emotion regulation in L2 acquisition primarily focus on strategies, categorized as downregulation and upregulation by Sutton and Harper (2009). Downregulation reduces negative emotions, while upregulation enhances positive ones. These strategies are widely used by L2 learners in classroom interactions and throughout the learning process (Gumora and Arsenio, 2002). Upregulating positive emotions enhances engagement, while downregulating negative emotions mitigates undesirable influences on language performance (Greenier et al., 2021; Teng and Zhang, 2018).

Gross (2015) distinguishes between extrinsic and intrinsic emotion regulation. Intrinsic regulation involves controlling one’s own emotions, whereas extrinsic regulation involves attempting to control another’s emotions. L2 students can use intrinsic regulation strategies such as deep breaths, positive reframing, and focusing on progress to maintain motivation despite difficulties. Cognitive reappraisal and expressive suppression are also commonly studied L2 emotion regulation strategies (Gross, 1998). The former is an adaptive emotion regulation strategy, where individuals adjust their thoughts about a situation to change its emotional impact (Zhou et al., 2023). In contrast, expressive suppression refers to the behavioral inhibition of emotional expression in social contexts (Polizzi and Lynn, 2021).

Students with greater emotional and cognitive competencies are better able to regulate and adapt to challenging language learning situations and ultimately improve their performance (Gumora and Arsenio, 2002; Teng and Zhang, 2018; Theiyab Alazemi et al., 2023). Effective emotion regulation helps learners manage the emotional challenges of L2 learning, such as frustration with language barriers or anxiety in speaking situations, leading to more positive learning outcomes.

Domestic studies have identified high-frequency emotion regulation strategies associated with capacity development (Xu and He, 2021; Xue and Wang, 2022). Xue and Wang (2022) categorize Chinese non-English major undergraduates’ emotion regulation in L2 contexts into excessive, low, and moderate regulation based on latent profile analysis. These findings highlight the importance of emotion regulation in L2 learning and its impact on students’ intellectual and emotional development.

### Resilience

In recent years, resilience has emerged as a pivotal concept in the study of well-being, particularly in the context of overcoming severe or prolonged adversities (Rutter, 2006). Originally defined as the ability to recover swiftly from setbacks, resilience research in psychology began in the 1970s, drawing from findings in progressive psychology and psychiatry (Luthar, 2006). Early research focused on children’s capacity to grow despite hardship, identifying protective traits that mitigate adversity’s negative impacts (Garmezy, 1974; Luthar and Cicchetti, 2000).

Over time, definitions of resilience have evolved, with Connor and Davidson (2003) emphasizing the personal qualities that enable individuals to thrive in the face of adversity. Luthar (2006) proposed a broader definition, highlighting the processes, capabilities, and outcomes of effective adjustment in challenging situations. In educational contexts, resilience is crucial for students facing various stressors, such as linguistic difficulties, cultural adaptation, and academic demands in second language (L2) learning environments (Dewaele et al., 2019; Masten, 2001). Resilient students manage challenges better than their peers, enhancing their ability to overcome adversity through a positive outlook (Martin and Marsh, 2006).

Educational resilience, providing a useful framework for understanding students’ adaptive capacities in learning contexts, comprises three components: negative consequences and emotional reactions, reflective thinking and seeking help, and persistence (Cassidy, 2015). Persistence involves tenacity and diligent approaches to academic challenges, while reflective thinking enables learners to adaptively seek assistance based on their abilities. Resilient learners exhibit strong interpersonal skills, high aspirations, a positive school attitude, self-esteem in academic abilities, and respect for institutional culture (Liu et al., 2022).

For empirical measurement, however, this study employs the Connor-Davidson Resilience Scale (CD-RISC; Connor and Davidson, 2003). The CD-RISC has been extensively validated across cultures, including in China, and provides a concise, psychometrically robust tool. Its components personal competence and tenacity, trust in instincts and stress tolerance, positive acceptance of change, sense of control, and spiritual influences. It aligns closely with key features of educational resilience, such as persistence, adaptive problem-solving, and supportive interpersonal engagement (Lyons, 1991; Rutter, 1985). Therefore, while educational resilience informs the conceptual understanding of student adaptation, CD-RISC allows reliable and culturally appropriate measurement in this study.

The second language acquisition process is often laborious and time-consuming, necessitating resilience to achieve educational goals under adverse conditions (Richardson, 2002). Researchers have outlined stages in the resilience process, including identifying traits that help overcome challenges, adapting by cultivating these traits, and overcoming obstacles to build a stronger resolve. Studies have underscored resilience’s relevance to engagement, well-being, and learning motivation in L2 contexts (Liu et al., 2022; Wang and Liu, 2022). By understanding and fostering resilience, educators can support L2 learners in navigating the complexities and challenges of their educational journeys, ultimately promoting their well-being and academic success.

### Studies on the relationship between well-being, emotion regulation, and resilience

Research consistently demonstrates emotion regulation’s pivotal role in enhancing well-being through positive emotion cultivation (Polizzi and Lynn, 2021), which in turn positively influences the well-being of second language (L2) learners. Maintaining both physical and mental well-being is crucial for overall health (Braunstein et al., 2017) and constitutes a fundamental aspect of social and emotional strategies in language learning (Liu et al., 2021). Effective emotion regulation has been shown to foster positive emotions, thereby promoting well-being. For instance, Shafiee Rad and Jafarpour (2023) found that positive emotional interventions have a favorable and significant impact on L2 learners’ writing skills and overall well-being. Their investigation into the effects of emotion regulation strategies revealed a significant enhancement in the experimental group’s well-being. Chen et al. (2022) further examined the indirect influence of emotion regulation on happiness through interpersonal relationships, underscoring the importance of integrating emotion regulation strategies in language learning for optimal outcomes.

Emotion regulation has been identified as a strong predictor of well-being (Li and Lv, 2022). A cross-sectional study by Shafiee Rad and Hashemian (2022), involving 312 students from five English language institutes in Iran, identified emotion regulation as a predictor of L2 hedonic orientations. Similarly, Chai et al. (2018) conducted research in China using the Emotion Regulation Scale (Gross and John, 2003) to investigate the impact of emotion regulation strategies on the well-being of migrant children. They found that the cognitive reappraisal technique, a specific emotion regulation strategy, had a direct and positive effect on these children’s well-being, consistent with previous studies (Gross and John, 2003). Expression suppression, however, was not found to be significant. Specifically, cognitive reappraisal emerged as an effective strategy for regulating the emotions of migrant children, leading to improved well-being.

Resilience, another critical factor, also positively impacts well-being (Johnson et al., 2016). Studies have shown that resilient individuals can protect themselves from the adverse effects of adversity and enhance their psychological well-being, thereby preserving their overall health (Connor and Davidson, 2003). Consequently, resilience is recognized as a significant contributor to well-being (Bajaj and Pande, 2016). For instance, during the COVID-19 pandemic, Renati et al. (2023) found that resilience mediated, either totally or partially, the connections between personality traits, well-being, and stress. Similarly, Wang et al. (2021) conducted a study revealing that children with high levels of resilience were more likely to experience happiness, success, and satisfaction with their lives. In the context of L2 learning, L2 learners with high degrees of resilience are more likely to experience greater joy, performance, and well-being (Wang and Liu, 2022).

Researchers have also focused on the interaction between emotion regulation and resilience, as both factors can significantly influence one’s sense of well-being. On the one hand, according to Li (2023) and Li and Lv (2022), emotion regulation is a good reflection of resilience. Individuals with high emotion regulation skills utilize positive emotion regulation strategies, which activate internal resources to stabilize emotional states, create positive factors, and increase resilience in the face of difficulties and adversity (Quoidbach et al., 2015). This, in turn, leads to well-being. The significant positive correlation between emotion regulation (specifically cognitive reappraisal) and resilience is exemplified in the research by Chai et al. (2018).

On the other hand, resilience plays a crucial role in various interaction processes, such as mediating the relationship between motivation and well-being (Wang and Liu, 2022) and expressing emotions and subjective well-being (Eldeleklioğlu and Yıldız, 2020). Yurtsever Gurkan (2025) revealed the effects of cognitive reappraisal strategies and resilience on the emotional well-being of medical students and the mediating effect of resilience in the relationship between positive reappraisal strategies and well-being. Similarly, Bajaj and Pande (2016) explored the mediating role of resilience in the impact of mindfulness on life satisfaction and affect, using 327 Indian university students. They found that resilience partially mediated the impact of mindfulness on life satisfaction and affect, highlighting the importance of resilience in understanding the beneficial effects of mindfulness on subjective well-being. Additionally, in the context of the impact of emotional intelligence on psychological well-being, resilience serves as a mediator (Akbari and Khormaiee, 2015). Their study with 405 high school students in Shiraz revealed resilience as a partial mediator, suggesting that emotional intelligence directly impacts psychological well-being and indirectly enhances it by fostering resilience.

In conclusion, the interplay between well-being, emotion regulation, and resilience is complex and multifaceted. Although research has investigated the correlation among these three factors (Eldeleklioğlu and Yıldız, 2020; Shafiee Rad and Hashemian, 2022; Shafiee Rad and Jafarpour, 2023), there is a scarcity of studies in the field of second language acquisition. This study aims to address this gap by examining these interactions and their combined effects on L2 learners’ well-being. Positive emotions and well-being are crucial in enhancing L2 learning experiences and outcomes. Emotion regulation and resilience are pivotal in managing the emotional challenges associated with second language acquisition. This study contributes to the understanding of how these factors interplay to influence L2 learners’ well-being, supporting the integration of emotion regulation strategies in language education to foster resilience and promote positive learning environments.

## Methods

### Participants

The participants were 157 sophomore students studying English language as an L2 (150 valid cases) from a top university in Beijing, China, including both genders (male = 68, female = 82). Their ages ranged from 19 to 21 years old, with an average age of 20. The pilot involved 34 senior students from a university in Beijing. Informed consent and willingness to participate in this study were obtained from all 191 participants before voluntarily participating via Wenjuanxing, an online questionnaire platform, utilizing convenience sampling.

### Instruments

#### PERMA well-being scale

Seligman (2011) delineates well-being through the PERMA model, comprising positive emotion, engagement, relationships, meaning, and achievement. Utilizing the PERMA scale created by Butler and Kern (2016) to generally assess participants’ well-being, the researchers assessed Martin Seligman’s five facets of well-being, encompassing 15 elements, with three in each aspect. Inquiries, as an 11-point Likert-type questionnaire, were graded on a scale of 0 to 10, where 0 signified extremely low levels, and 10 denoted extremely high levels. The reliability and validity of the PERMA-Profiler subscales were validated to be satisfactory by Butler and Kern (2016). The present study adopted a 5-point Likert scale, and the Cronbach’s Alpha of the pilot was 0.95.

#### Emotion regulation questionnaire

Previous studies (Greenier et al., 2021; Shafiee Rad and Jafarpour, 2023) frequently utilized emotion regulation strategy scales to assess emotion regulation. Consequently, this study utilized the emotion regulation questionnaire developed by Gross and John (2003) to analyze participants’ emotion regulation. Ten items and two dimensions were used to gauge the respondents’ emotion regulation. Cognitive reappraisal (e.g., “I control my emotions by changing the way I think about the situation I am in”) and expressive suppression (e.g., “When I am feeling negative emotions, I make sure not to express them”) were assessed in the first and second sections, respectively. The questionnaire was based on a Likert-type 7-point scale, where 1 represented strongly disagree and 7 represented strongly agree. It should be mentioned that Cronbach’s Alpha determined the scale’s reliability and internal consistency, and the result was 0.93 (Shafiee Rad and Jafarpour, 2023). In this study, a 5-point Likert scale was employed. The Cronbach’s Alpha coefficient obtained from the pilot test reached 0.83.

#### Connor-Davidson resilience scale

The Connor-Davidson Resilience Scale (CD-RISC), developed by Connor and Davidson (2003), was a 25-item assessment tool on a 5-point Likert scale to gauge an individual’s resilience to adversity. This study employed the 10-item form of the measure (Campbell-Sills and Stein, 2007). With a Cronbach’s Alpha of 0.93, this scale demonstrated strong construct validity and internal consistency (Campbell-Sills and Stein, 2007), ensuring sufficient measure reliability. It contained qualities like “not easily discouraged by failure,” “able to adapt to change,” and “can stay focused under pressure.” A five-point Likert scale, ranging from 1 to 5, was used to rate the items in this study. Greater resilience was correlated with higher scores. The assessed Cronbach’s Alpha of the pilot was 0.93.

### Data collection procedures

To meet the objectives of the study, the package of three scales on PERMA well-being, emotion regulation (cognitive reappraisal and expressive suppression), and resilience was distributed to 34 students via the online questionnaire platform Wenjuanxing as a pilot. The researchers conducted a Cronbach’s Alpha test for each questionnaire based on data collected from the pilot. Based on the pilot study results, the researchers modified the questionnaires in three ways. First, items were rephrased to better suit the context of L2 learning. For example, the original item “How satisfied are you with your personal relationships?” was changed to “How satisfied are you with your personal relationships in English language learning journey.” This adjustment was made to make the items more relevant and easier for L2 learners to understand. Second, in the Emotion Regulation Questionnaire, the researchers simplified the language for better comprehension by L2 learners. The original statement “When I want to feel less negative emotion, I change the way I’m thinking about the situation” was rewritten as “When I do not want to feel bad, I try to think about the situation differently.” Finally, in order to reduce their cognitive burden, the researchers reduced the response format from the original Likert scale to a 5-point Likert version, which pilot testing demonstrated was more discriminable for L2 learners while maintaining acceptable reliability (Cronbach’s Alpha > 0.8). As Hinkin (1995) suggested, scale adaptations were sometimes necessary to enhance respondent comprehension and reduce measurement error.

The formal data were collected through Wenjuanxing, an online questionnaire platform, at the end of February 2024. Prior to participation, all participants were presented with an online informed consent form, which detailed the study’s purpose, procedures, potential risks and benefits, and the confidentiality of their data. They were informed that their participation was voluntary and that they could withdraw at any time without penalty. Only those who provided consent could access the questionnaires. All participants were informed of how to fill in the self-report scale and were assured that their responses and personal information would only be used for research purposes and would remain confidential. For the entire duration of the data collection process, the researchers strictly adhered to the ethical principles throughout the entire process.

### Data analysis

The data were analyzed using SPSS and the SPSS PROCESS macro. Prior to formal analysis, several preliminary steps were undertaken. The reliability and validity of the measurement instruments were assessed using Cronbach’s Alpha and the KMO test, respectively. Additionally, the normality of the data distribution for all key variables was examined using the Shapiro–Wilk test to justify the use of parametric tests.

Although emotion regulation was often conceptualized as a component of resilience (Braunstein et al., 2017), in this study, the two constructs were treated as distinct. Separate validated instruments were employed to measure them, enabling us to examine their unique as well as interdependent effects on well-being. In this study, emotion regulation was divided into two sub-dimensions: cognitive reappraisal and expressive suppression. To address the research questions, Pearson correlation analysis was first conducted to calculate correlation coefficients between emotion regulation, resilience, and well-being. A nested and hierarchical multiple regression with partial F test was conducted for the second research question, which intended to examine the predictor role of cognitive reappraisal and expressive suppression in L2 learners’ well-being. Finally, to examine whether resilience mediates the prediction of cognitive reappraisal and expressive suppression on well-being, mediation analyses were conducted using Model 4 of Hayes’ PROCESS macro for SPSS (Hayes, 2013). This model, which estimates simple mediation models with one mediator, also calculates both direct and indirect effects, provides bootstrapped confidence intervals, and is widely applied in psychology and social sciences to test mediation hypotheses.

## Results

To ensure the reliability and validity of the instruments used for data collection, the researchers ran Cronbach’s Alpha test, KMO test, and Bartlett’s test for each questionnaire. Table 1 showed that all three questionnaires, including PERMA well-being, emotion regulation (cognitive reappraisal and expressive suppression), and resilience, had satisfactory Cronbach’s Alpha indices (0.948, 0.908, 0.851, and 0.913, respectively). The KMO test yielded a value of 0.926, 0.906, 0.809 and, 0.919, indicating sufficient sampling adequacy. Moreover, Bartlett’s test showed a significant result (*p* < 0.01), confirming non-sphericity and supporting the feasibility of conducting factor analysis.

**Table 1:** Reliability of the questionnaires.

Scales		Cronbach’s Alpha coefficient	Number of items
Well-being		0.948	1–15
Emotion regulation	Cognitive reappraisal	0.908	16, 18, 20, 22, 23, 25
	Expressive suppression	0.851	17, 19, 21, 24
	The whole	0.913	16–25
Resilience		0.913	26–35

After ensuring the reliability of the questionnaires, the researchers conducted a normality test to decide whether the data should be analyzed parametrically. Given that the sample size was less than 5,000, the Shapiro–Wilk test was used. The collected data exhibited a normal distribution for well-being and resilience, as their significance values were above 0.05. Despite the Shapiro–Wilk test results showing significance levels below 0.05 for cognitive reappraisal (skewness = −0.215, kurtosis = −0.915) and expressive suppression (skewness = 0.304, kurtosis = −0.712) variables, their distributional properties suggested approximate normality. Descriptive statistics indicated that the absolute values of kurtosis and skewness were below 10 and 3, respectively. Bell-shaped normal histograms further supported this. Thus, although not strictly normal, the data could be treated as approximately normal for parametric analysis.

Consequently, parametric statistical methods could be reliably employed to analyze the data, ensuring the validity of subsequent statistical inferences.

Correlation analysis was used to answer the first research question, and the results were shown in Table 2.

**Table 2:** The Pearson correlation matrix among variables.

Scales		Well-being	Emotion regulation		Resilience
			Cognitive reappraisal	Expressive suppressing	
Well-being		1			
Emotion regulation	Cognitive reappraisal	0.706**	1		
	Expressive suppression	0.564**	0.618**	1	
Resilience		0.832**	0.722**	0.613**	1

**p < 0.01.

Pearson correlation coefficient showed the strength and direction of the relationship among the variables. Table 2 demonstrated that the relationship between well-being and the other variables (cognitive reappraisal, expressive suppression, and resilience) was direct (0.706, 0.564, 0.832), which meant that the higher index of well-being, the higher indices of the other variables. A positive correlation coefficient of 0.618 existed between cognitive reappraisal and expressive suppression. Among L2 learners preferring cognitive reappraisal, expressive suppression was still frequently used, suggesting the coexistence of these two emotion regulation strategies. Furthermore, the significance level for all these relationships was below 0.01, which meant that there was a direct and significant relationship between cognitive reappraisal, expressive suppression, resilience, and well-being among L2 learners.

To answer the second research question, which concerned whether L2 learners’ emotion regulation (cognitive reappraisal and expressive suppression) in this study significantly predicted their well-being, the researchers ran a nested and hierarchical multiple regression with theory-driven blocks. Incremental variance explained by each step was evaluated via the partial F test (F-change). Model 1 included only cognitive reappraisal to assess its predictive power for well-being. Model 2 added expressive suppression to examine its unique increment beyond cognitive reappraisal.via partial F. The interaction term (cognitive reappraisal * expressive suppression) were added in Model 3 to test a potential effect and its incremental variance with the Enter method. Model assumptions were checked and met because multicollinearity was negligible (all VIF ≤ 1.70; see Table 3) and residual independence was acceptable (Durbin-Watson = 1.885; see Table 4).

**Table 3:** Coefficients for cognitive reappraisal, expressive suppression and well-being.

Model		Unstandardized coefficients		Standardized coefficients	*T*	Sig.	VIF
		*B*	Std. error	Beta			
1	Constant	1.038	0.191	–	5.442	<0.001	–
	Cognitive reappraisal	0.658	0.054	0.706	12.136	<0.001	1.000
2	Constant	0.884	0.194	–	4.557	<0.001	–
	Cognitive reappraisal	0.540	0.067	0.579	8.009	<0.001	1.617
	Expressive suppression	0.188	0.066	0.206	2.850	0.005	1.617
3	Constant	0.907	0.198	–	4.577	<0.001	–
	Cognitive reappraisal	0.531	0.069	0.569	7.676	<0.001	1.696
	Expressive suppression	0.196	0.067	0.215	2.906	0.004	1.693
	Interaction	−0.030	0.049	−0.035	−0.597	0.551	1.059

Dependent variable: well-being.

**Table 4:** Summary of the regression model.

Model	*R*	*R* ^2^	Adj *R*^2^	Std. error	*R*^2^ change	*F* change	df1	df2	*p* (F change)	Durbin-Watson
1	0.706^a^	0.499	0.495	0.659	0.499	147.279	1	148	<0.001	
2	0.725^b^	0.525	0.519	0.644	0.026	8.124	1	147	0.005	
3	0.725^c^	0.526	0.516	0.645	0.001	0.357	1	146	0.551	1.885

^a^Predictors: (constant), cognitive reappraisal. ^b^Predictors: (constant), cognitive reappraisal, expressive suppression. ^c^Predictors: (constant), cognitive reappraisal, expressive suppression, interaction. ^d^Dependent variable: well-being.

Table 4 summarized the regression model and partial F tests. Model 1, with cognitive reappraisal only, was significant with substantial explanatory power (*R* = 0.706, *R*^2^ = 0.499) indicating that cognitive reappraisal alone explains about 49.9% of the variance in well-being. Adding expressive suppression (Model 2) increased *R*^2^ to 0.525, yielding an additional variance was 0.026 (from 0.499 to 0.525) and representing a 2.6% increase in explanatory power. It was significant for F-change (1,147) = 8.124, *p* = 0.005. The partial F test therefore demonstrated that expressive suppression contributed a small but significant unique increase beyond cognitive reappraisal. By contrast, adding the interaction term in Model 3 did not significantly improve the model (F-change (1,146) = 0.357, *p* = 0.551), indicating that the effects of cognitive reappraisal and expressive suppression on well-being were primarily additive rather than interactive.

Table 5 examined the statistical significance of the three regression models. Model 1, *F*(1,148) = 147.279, *p* < 0.001; Model 2, *F*(2,147) = 81.246, *p* < 0.001; and Model 3, *F*(3,146) = 54.046, *p* < 0.001. All models were statistically significant, indicating good overall model fit. However, as emphasized in hierarchical regression literature (Cohen et al., 2003), the omnibus F test cannot determine whether newly added predictors provide unique contributions. Therefore, we relied on the partial F test to evaluate the incremental validity of expressive suppression and the interaction term (Petrocelli, 2003). The significant F-change from Model 1 to 2 justified including suppression, whereas the non-significant F-change from Model 2 to 3 suggested that the interaction was not meaningful. Thus, the combined effects in Model 2 offered the best balance of parsimony and explanatory power.

**Table 5:** ANOVA^a^ for regression.

Model		Sum of squares	df	Mean square	*F*	Sig.
1	Regression	63.942	1	63.942	147.279	<0.001^b^
	Residual	64.255	148	0.434	–	–
	Total	128.197	149	–	–	–
2	Regression	67.307	2	33.653	81.246	<0.001^c^
	Residual	60.890	147	0.414	–	–
	Total	128.197	149	–	–	–
3	Regression	67.455	3	22.485	54.046	<0.001^d^
	Residual	60.741	146	0.416	–	–
	Total	128.197	149	–	–	–

^a^Dependent variable: well-being. ^b^Predictors: (Constant), cognitive reappraisal. ^c^Predictors: (Constant), cognitive reappraisal, expressive suppression. ^d^Predictors: (constant), cognitive reappraisal, expressive suppression, interaction.

Finally, the regression coefficients for the model were reported in Table 3.

In Model 2, cognitive reappraisal showed a strong positive effect (Beta = 0.579, *t* = 8.009, *p* < 0.001), while expressive suppression showed a smaller but significant positive effect (*β* = 0.206, *t* = 2.850, *p* = 0.005). To compare the strength of predictors, we focused on the standardized coefficients (Beta) rather than unstandardized coefficients, since they are placed on the same scale. These results indicated that cognitive reappraisal was a substantially stronger predictor of well-being than expressive suppression, though both were significant.

The final regression equation, based on Model 2, was expressed as:


$$\begin{array}{l} \text{Well}-\text{being}=0.884+0.54^{*}\;\text{Cognitive reappraisal}+\\ 0.188^{*}\;\text{Expressive suppression} \end{array}$$

Therefore, both cognitive reappraisal and expressive suppression significantly and positively predicted well-being among L2 learners, with cognitive reappraisal exerting a much stronger effect. These findings underscored the role of effective emotion regulation strategies, particularly cognitive reappraisal, as critical predictors of well-being.

This study employed Hayes (2013) Process Plugin Model 4, which estimated simple mediation models with one mediator, to investigate the mediating role of resilience. The researchers conducted mediation analyses using cognitive reappraisal and expressive suppression as independent variables, respectively.

Table 6 presented the regression results for cognitive reappraisal, resilience, and well-being. Without considering resilience as a mediator, the path coefficient for cognitive reappraisal predicting well-being was 0.658 (*β* = 0.658, *t* = 12.136, *p* < 0.001). When resilience was introduced as a mediator, the path coefficient for resilience predicting well-being was 0.761 (*β* = 0.761, *t* = 10.563, *p* < 0.001), and the direct effect of cognitive reappraisal on well-being remained significant (*β* = 0.206, *t* = 3.479, *p* < 0.001). This indicated that resilience partially mediates the relationship between cognitive reappraisal and well-being.

**Table 6:** Regression of mediating model.

Mediator	Model	Outcome variable	Predictor variable	*R*	*R* ^2^	*F*	*β*	*T*	Sig.
Cognitive reappraisal	1	Well-being	Cognitive reappraisal	0.706	0.499	147.279	0.658	12.136	0.000
	2	Resilience	Cognitive reappraisal	0.722	0.521	160.940	0.594	12.686	0.000
	3	Well-being	Cognitive reappraisal	0.846	0.715	184.447	0.206	3.479	0.000
			Resilience	–	–	–	0.761	10.563	0.000
Expressive suppression	1	Well-being	Expressive suppression	0.564	0.318	68.537	0.513	8.303	0.000
	2	Resilience	Expressive suppression	0.613	0.376	89.140	0.493	9.441	0.000
	3	Well-being	Expressive suppression	0.834	0.696	168.472	0.079	1.499	0.136
			Resilience	–	–	–	0.882	13.534	0.000

As shown in Table 7, both the direct effect of cognitive reappraisal on well-being and resilience’s mediating effect were statistically significant, as their 95% confidence intervals excluded zero (Table 7).

**Table 7:** Total, direct, and indirect effects on well-being.

Mediator	Effect type	Effect	BootSE	BootLLCI	BootULCI	Relative effect size
Cognitive reappraisal	Total effect	0.658	0.054	0.551	0.766	100%
	Direct effect	0.206	0.059	0.089	0.323	31.3%
	Indirect effect	0.452	0.070	0.320	0.592	68.7%
Expressive suppression	Total effect	0.513	0.062	0.391	0.635	100%
	Direct effect	0.079	0.052	−0.025	0.182	15.2%
	Indirect effect	0.435	0.058	0.325	0.550	84.8%

Therefore, resilience played a partial mediating role in predicting well-being through emotion regulation, with a mediating effect of 0.452, accounting for 68.7% of the total effect.

**Figure 1:**
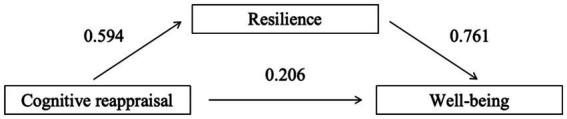
Mediation path coefficient of cognitive reappraisal, resilience, and well-being.

The mediation path of the model was demonstrated in Figure 1.

Tables 6, 7 presented the complete mediation effect with expressive suppression as the independent variable. Table 6 showed that after including resilience as a mediator, the direct effect of expressive suppression on well-being was no longer significant (*β* = 0.079, *t* = 1.499, *p* = 0.136) with a relative effect size of 84.8%, indicating resilience fully mediated its relationship with well-being.

**Figure 2:**
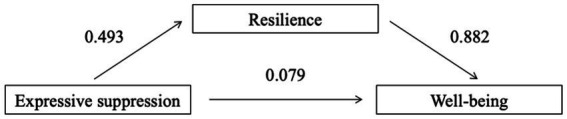
Mediation path coefficient of expressive suppression, resilience, and well-being.

The mediation path of the model was demonstrated in Figure 2.

In conclusion, the above results verified the mediational effect of resilience in the predictive role of emotion regulation (cognitive reappraisal and expressive suppression) on well-being. Cognitive reappraisal not only exerted direct effects on well-being but also indirectly impacted it by enhancing resilience. In contrast, the impact of expressive suppression on well-being was completely realized through resilience. These results highlighted the potential importance of targeting both emotion regulation and resilience to promote the well-being of L2 learners.

## Discussion

This study reveals complex relationships between resilience, emotion regulation (comprising cognitive reappraisal and expressive suppression), and well-being among L2 learners, with each pair showing positive correlations. Specifically, learners with superior emotion regulation skills tend to exhibit heightened resilience and well-being, while those with weaker emotion regulation abilities display lower levels of resilience and well-being. A relatively high correlation exists between cognitive reappraisal and expressive suppression, indicating their co-occurrence. These results underscore the substantial and meaningful association among these variables.

The results yield two significant insights: (1) cognitive reappraisal enhances well-being both directly and indirectly through resilience, and (2) expressive suppression’s impact on well-being is entirely mediated by resilience. These findings offer valuable insights into the psychological processes that shape well-being among L2 learners, emphasizing the importance of adaptive emotional strategies in fostering positive learning experiences. Notably, a gap in the existing literature pertains to the lack of research examining these relationships specifically among Chinese L2 learners.

First, between emotion regulation and well-being, learners experiencing higher levels of well-being typically possess superior emotion regulation skills. This connection emphasizes the pivotal role of emotional factors in language learning and underscores the potential benefits of fostering positive well-being to enhance emotion regulation abilities among language learners. Effectively managing both positive and negative emotions fosters a conducive learning environment, which, in turn, promotes well-being. Consistent with existing literature (Wang et al., 2025), well-being shows a particularly strong association with cognitive reappraisal, a strategy known to foster positive emotionality. In L2 learning contexts, cognitive reappraisal enables learners to reframe challenges positively; for example, interpreting pronunciation errors as improvement opportunities rather than failures. This adaptive reinterpretation modulates emotional responses and builds resilience against L2 learning stressors, ultimately enhancing overall well-being.

Notably, while expressive suppression is typically considered a maladaptive strategy (Johnson et al., 2016), in the L2 learning context in China, learners with an effective strategy of expressive suppression strategies may also have a high level of well-being. This context-specific finding suggests that suppressing negative emotional displays may contribute to maintaining harmonious classroom dynamics and facilitate objective self-assessment of progress (Namaziandost et al., 2023), indirectly supporting well-being. Furthermore, well-being positively influenced learners’ metacognitive awareness and self-monitoring capacities.

Butler et al. (2007) argue that in collectivist cultures, suppression may not carry the same interpersonal costs as in individualistic societies because harmony preservation is strongly valued. This perspective helps explain why our participants could benefit from suppression when it was buffered by resilience. In the Chinese classroom context, students are often encouraged to prioritize group cohesion, maintain respectful silence, and demonstrate deference to teachers. Within this cultural and educational environment, expressive suppression may serve as a socially adaptive behavior that aligns with classroom norms. When supported by resilience, such suppression does not lead to psychological harm but instead facilitates smoother adjustment to academic demands.

Secondly, between emotion regulation and well-being, effective emotion regulation is crucial for managing language learning stresses, while resilience enables learners to overcome setbacks and maintain motivation, effort and engagement during challenging learning experiences. Multiple studies have indicated that cognitive reappraisal is positively associated with resilience, while expressive suppression is negatively related (Shafiee Rad and Jafarpour, 2023). However, this study demonstrates positive associations between both cognitive reappraisal and expressive suppression with L2 learners’ resilience. This dual-pathway relationship suggests context-specific mechanisms in language learning environments. Cognitive reappraisal enhances resilience by enabling learners to reinterpret stressors, thereby reducing their emotional impact. Conversely, expressive suppression, typically associated with negative outcomes like increased rumination (Johnson et al., 2016), also showed resilience benefits in our L2 context. Therefore, both cognitive reappraisal and expressive suppression can contribute to decreasing the stress level and then increase L2 learners’ resilience when navigating difficult linguistic tasks and unfamiliar cultural contexts. Learners adept at emotion regulation utilize positive strategies as protective factors, stabilizing emotional states and enhancing resilience (Martínez-Priego et al., 2024). This regulation fosters persistence and motivation, enabling learners to rebound from setbacks, engage actively in learning, build relationships, and gain accomplishment (Namaziandost et al., 2023).

Thirdly, between well-being and resilience, learners who report higher levels of well-being exhibit greater resilience when confronted with challenges. This connection underscores the pivotal role of positive psychological states in fostering adaptive responses to language learning obstacles, which is essential for achieving success and satisfaction in acquiring a new language. Resilient L2 learners experience higher levels of happiness, performance, and overall well-being (Wang and Liu, 2022). These results align with those of Shafiee Rad and Jafarpour (2023), who similarly found a significant positive correlation between resilience and L2 students’ well-being. Resilient individuals manage stress effectively, navigating anxieties and embracing linguistic complexities with flexibility, mitigating frustration. Their optimistic mindset views setbacks as transient obstacles, fostering perseverance, motivation, and sustained engagement, thereby enhancing well-being. This ability to regulate stress cultivates an environment conducive to maintaining well-being throughout the learning process.

This study aligns with prior research demonstrating interrelationships among emotion regulation, resilience, and well-being (Chai et al., 2018; Eldeleklioğlu and Yıldız, 2020; Wang et al., 2021). Our analysis confirms that these variables are similarly interrelated among Chinese L2 learners, corroborating recent Chinese studies (Li, 2023; Li and Lv, 2022). Through emotion regulation, learners manage stress and obstacles by effectively assessing and modifying emotions, thereby enhancing motivation, satisfaction, and comfort in learning, ultimately increasing well-being.

Most importantly, this study demonstrates two mediation pathways in the Chinese L2 learning context. These results are intriguing, as they suggest that different emotion regulation strategies have distinct pathways through which they influence L2 learners’ emotional outcomes. Specifically, cognitive reappraisal has a partial mediating effect on the relationship between emotion regulation and well-being, while expressive suppression affects well-being only through its full mediation by resilience. However, previous research found that both cognitive reappraisal and expressive suppression significantly and positively predict well-being (Gross and John, 2003). This highlights suppression’s unique role in L2 learning contexts in China.

Learners who regulate emotions effectively experience more positive emotions and are less likely to be overwhelmed by negative ones, leading to greater overall life satisfaction and a sense of well-being. Emotion regulation optimizes cognitive (attention, memory, problem-solving) and expressive processes crucial for language acquisition (Namaziandost et al., 2023). It suggests that heightened emotional arousal, whether positive or negative, can impact cognitive functioning, including attention, memory, and problem-solving abilities, but also affect communicative expression, including concealing emotions from others.

Cognitive reappraisal, which involves changing the way one thinks about a situation to alter its emotional impact, directly predicts well-being. This direct effect aligns with prior research (Li, 2023) and extends the findings of Gross and John (2003), as reinterpreting negative situations as opportunities for growth leads to more positive emotional experiences. In the context of L2 learning, cognitive reappraisal enables learners to reinterpret stressful language learning tasks, such as struggling with pronunciation or grammar mistakes, as opportunities to improve rather than as failures. This positive reframing reduces emotional stress and enhances overall well-being.

The study also finds that resilience partially mediates the relationship between cognitive reappraisal and well-being. This suggests that cognitive reappraisal not only directly improves emotional outcomes but also helps learners develop resilience. When learners reframe challenges as learning opportunities, they are better equipped to cope with future stressors and setbacks, such as language anxiety or performance pressure, thereby increasing their emotional resilience. This mediating effect of resilience is crucial because resilient learners tend to bounce back from emotional setbacks more effectively, maintaining their motivation and engagement in the learning process (Ge, 2025).

For educators and practitioners, fostering cognitive reappraisal could be a key intervention to enhance learners’ emotional resilience. By teaching L2 learners to reinterpret stressful situations more positively, educators can help them build both emotional regulation skills and resilience, thus promoting their overall well-being.

On the other hand, expressive suppression, typically considered a maladaptive emotion regulation strategy, was found to be beneficial only when mediated by resilience. This divergence highlights suppression’s unique role in L2 learning contexts. While suppressing emotions in L2 learning does not resolve emotional issues and can be burdensome, resilient learners can better manage the resulting distress from suppression and use it productively. A lack of resilience may lead to stress and decreased well-being. For instance, suppressing visible resistance during sensitive discussions maintains harmony. By modulating emotional responses, learners optimize resources for language tasks, leading to efficient learning and enhanced well-being.

In the Chinese education system, exams are extremely important, as they serve as the determining factor for students’ access to higher education and future opportunities. Notably, such high-stakes scenarios, like language exams or presentations, often intersect with a key psychological phenomenon: expressive suppression, which involves inhibiting emotional expression in social contexts (Gross and John, 2003). For example, in high-stakes situations such as language exams or presentations, learners might suppress negative emotions like anxiety or fear to maintain composure. While this suppression does not directly improve well-being, it can prevent emotional outbursts and maintain focus on the task at hand. However, for this strategy to be beneficial, resilience is critical. Resilient learners are better able to handle the emotional toll of suppression, using it as a short-term coping mechanism without experiencing long-term negative effects on their well-being. Without resilience, expressive suppression could lead to emotional distress, increasing rumination or exacerbating stress (Johnson et al., 2016).

The full mediation of expressive suppression by resilience suggests that it is not the suppression itself that is beneficial, but rather the resilience that enables learners to manage and recover from the emotional consequences of suppressing their feelings. This finding highlights the importance of resilience as a protective factor in L2 learning. While expressive suppression may be an adaptive strategy in certain contexts, it should be used judiciously, and learners need to build resilience to mitigate any negative emotional impacts.

It is important to acknowledge that L2 learners often face identity-related challenges, especially those related to cultural adaptation and language proficiency. These identity issues can contribute to emotional stress, which in turn affects well-being. For example, learners who struggle with language proficiency may experience feelings of inadequacy or embarrassment, which can undermine their sense of identity and emotional well-being.

To address these identity-driven emotional stressors and protect learners’ well-being, the roles of resilience and emotion regulation become particularly critical, and it is valuable to examine how they operate. Specifically, L2 learners with emotional and cognitive competencies adeptly regulate and adapt their performance (Gumora and Arsenio, 2002), and emotion regulation, a key component of such emotional competencies, plays a central role in this process. Emotion regulation balances positive and negative motivations (Bielak and Mystkowska-Wiertelak, 2022), turning challenges into positive emotions, curbing demotivation, tempering excessive positivity, heightening engagement in activities and social interactions, and helping build relationships. Yet the ability to fully leverage these benefits of emotion regulation, especially amid L2 learning stress, depends heavily on learners’ resilience, and these outcomes directly underscore why resilience should be an integral focus in L2 education. Resilient learners, who can adapt to emotional stressors, tend to experience higher levels of engagement, motivation, and well-being. Interventions aimed at building resilience, such as mindfulness training, problem-solving exercises, and emotional support, can thus enhance the effectiveness of emotion regulation strategies like cognitive reappraisal and expressive suppression.

In conclusion, this study reveals that interventions improving emotion regulation skills may enhance well-being by bolstering resilience, through the partial or complete mediating role of resilience. This aligns with studies linking emotion regulation and resilience (Chai et al., 2018) and demonstrating resilience’s mediation between emotion regulation and engagement (Namaziandost et al., 2023). It also provides critical insights into whether their effects on well-being are additive, overlapping, or mediated. This joint investigation enables more precise identification of intervention targets, potentially maximizing the positive impact on L2 learners’ academic experiences.

## Conclusion and implications

This study significantly contributes to the literature on resilience, emotion regulation, and well-being among L2 learners, demonstrating their potential utility in fostering overall well-being. Key findings reveal positive correlations among these factors, with resilience showing the strongest link to well-being, followed by its correlation with emotion regulation. Cognitive reappraisal positively predicts well-being, with resilience partially mediating this relationship. Expressive suppression influences L2 learners’ well-being entirely through resilience.

From an instructional perspective, university teachers play a crucial role in cultivating emotion regulation and resilience. Mindfulness practices and a supportive classroom environment can create a conducive atmosphere for emotional well-being and academic success. Specifically, educators can implement brief cognitive reappraisal micro-lessons after challenging activities, guiding students to reframe negative thoughts (e.g., from “I failed that presentation” to “I’ve identified exactly what to practice next”). Regular self-reflection exercises within the curriculum can empower students to evaluate their emotional responses, recognize triggers, and develop effective strategies for regulating their emotions in diverse language learning contexts. Additionally, structured resilience journals enable learners to document challenges, emotional reactions, and coping methods. Workshops on resilience and positive thinking can further help students manage academic stress and navigate language learning difficulties.

Collaborative learning opportunities should also be expanded. Through cooperative exercises, teachers can facilitate problem-solving, idea-sharing, and peer feedback, which not only promotes resilience but also helps learners in regulating their emotions through social interaction. For instance, designing goal-oriented group projects (e.g., creating a podcast) forces students to solve problems collectively, thereby building resilience and providing a natural context for peer emotional support. Such dynamic classroom environments are crucial for nurturing a holistic approach to learning and well-being.

University administrators are vital in establishing a supportive ecosystem. By fostering open communication and empathy, they can help L2 learners feel safe expressing emotions and seeking help. Concrete measures include peer-mentoring programs linking novice and experienced learners, along with clear access to counseling psychological services tailored to academic stress. Such a framework reinforces both emotion regulation and resilience, enhancing student well-being.

Beyond the classroom, this study underscores the need for language programs that systematically integrate resilience and emotion regulation strategies. These may include mental health workshops, peer support groups, and counseling services. At the program level, these competencies should be embedded as explicit learning objectives; for instance, through a dedicated module on managing language anxiety, complete with targeted activities and assessments tracking both linguistic and emotional growth.

This study’s findings should be considered in light of its limitations, which in turn suggest avenues for future research. The reliance on a homogenous sample of sophomore L2 English learners from a single university restricts the generalizability of the results; therefore, future work should recruit more diverse participants from various geographical, educational, and linguistic backgrounds. Notably, when expanding the participant pool, it is essential to balance diversity and convergence (Li et al., 2025). Furthermore, while our quantitative, cross-sectional approach was informative, it could be enhanced by employing longitudinal or mixed-methods designs. Incorporating qualitative tools such as interviews and journals would provide a more dynamic and nuanced understanding of the interplay between resilience, emotion regulation, and well-being. Additionally, future studies would benefit from including objective academic outcomes (e.g., proficiency scores, GPA) to examine the critical well-being-achievement relationship. Finally, the theoretical model could be extended by testing emotion regulation as a potential mediator between resilience and well-being to further elucidate the underlying psychological mechanisms.

In conclusion, this study underscores the importance of resilience and emotion regulation in fostering well-being among L2 learners. By integrating these strategies into educational policies, classroom instruction, and learner support, we can create more supportive and effective learning environments that may promote holistic development and academic success. Future research should continue to explore this area, employing diverse methodologies, samples, and variables to enhance our understanding and inform practice.

## Data Availability

The original contributions presented in the study are included in the article/supplementary material, further inquiries can be directed to the corresponding author.
